# Infection prevention and control: Qualitative study of the preparedness and response of Christian health Association of Ghana to Marburg virus disease in Ghana

**DOI:** 10.1016/j.heliyon.2024.e31953

**Published:** 2024-05-25

**Authors:** Herman Nuake Kofi Agboh, George Adjeisah Adjei, Grace Adjei Okai, Caroline Awotwe, Benjamin Martey Ossom, Lily Yarney

**Affiliations:** a Catholic Health Service Trust, Ghana, P. O. Box 9712, Airport, Accra, Ghana; b University of Ghana Business School, P. O. Box LG 78, Legon, Accra, Ghana

**Keywords:** Preparedness, Emergency response, Disease surveillance, Infection prevention and control

## Abstract

**Objective:**

Recent disease outbreaks underscore the importance of robust disease surveillance and infection prevention and control (IPC) programmes to bolster Africa's public health response system. Yet, available evidence shows extensive gaps in the emergency response capacity of faith-based healthcare providers on the continent. Accordingly, this study examines the IPC and surveillance strategies adopted by a faith-based healthcare provider and the challenges encountered during Marburg Virus Disease outbreak (MVD) in Ghana.

**Method:**

We collected data from 15 clinical and nonclinical health workers from the Christian Health Association of Ghana (CHAG) and the Ghana Health Service (GHS). Data was collected through online interviews to examine two pillars of the WHO COVID-19 SPRP-AFR (2021) framework. We analyzed the data using Braun and Clarke's thematic analysis.

**Findings:**

The facility performed creditably well with contact tracing and other quarantine protocols during MVD outbreak in Ghana. However, it also encountered several challenges in the enforcement of IPC protocols, including human resource constraints, the lack of decontamination equipment, and limited infrastructure, among others. Given these limitations, we assessed that the facility cannot handle major outbreaks.

**Conclusion:**

Due to numerous infectious disease outbreaks in Sub-Saharan Africa in recent years, the government of Ghana and faith-based healthcare providers must resource their facilities with the relevant equipment and qualified human resources against future disease outbreaks.

## 1 Introduction

The effects of recent epidemics on life and commerce underscore the urgent need for effective health systems to mitigate infectious disease outbreaks in Africa [1]. In the last decade, for instance, West African countries faced outbreaks of Influenza, Ebola, COVID-19, and Marburg Virus Disease (MVD), among others [[1], [2], [3]]. Ebola outbreak between 2014 and 2016 claimed at least 11,325 lives in West Africa [1], while an estimated 250,000 African children aged zero to five are hospitalized annually due to influenza (H1N1, H7N9, H5N1) [4]. Additionally, Africa, with its limited health infrastructure and underdeveloped health systems, has been severely affected by the recent COVID-19 pandemic [5,6]. Inadequate public health interventions worsened the pandemic's impact, leading to increased transmissibility, reduced compliance with safety protocols, and inconsistent application of control measures at healthcare facilities. Since 1975, Africa experienced MVD outbreaks in seven countries, including South Africa (1975), Kenya (1980, 1987), the Democratic Republic of Congo (2000), Angola (2005), Uganda (2005, 2008, 2012, 2014, 2017), Guinea (2021), Ghana (2022) Equatorial Guinea (2023), and Tanzania (2023) [3]. Data from these outbreaks revealed a consistent pattern of weak health system response and high case fatality rates, ranging between 100 % in Guinea, Kenya, and Uganda to 33 % in South Africa [3].

MVD is a virulent disease transmitted through direct contact with infected blood, secretions, organs, and other bodily fluids. The virus can also be contracted through contact with infected surfaces and materials. Symptoms include haemorrhagic fever with a fatality ratio of approximately 88 % [7]. On July 7, 2022, the Ministry of Health (MoH) reported two MVD-positive cases in a faith-based healthcare facility in Ghana. The patients tested positive for Viral haemorrhagic fever and died within 24 h of admission. The overall case fatality rate in Ghana stood at 75 % [3]. Following the declaration, MoH, in collaboration with various partners, initiated infection prevention and control (IPC) measures to stem the disease's spread and associated fatalities. Strategies included collaborations with District Health Directorates, Noguchi Memorial Institute for Medical Research, and Senegal's Institut Pasteur for sample collection and testing. Partnerships were also formed with the Christian Health Association of Ghana (CHAG) to mobilize human resources for IPC and other public health interventions. The World Health Organization (WHO) supported joint surveillance teams for contact tracing and provided Personal Protective Equipment (PPE), alongside implementing quarantine and social distancing measures and other disease control strategies [8]. Although these efforts significantly reduced the number of infections and deaths from MVD, some criticized the response as reactionary [9,10].

Given the limited data on MVD outbreaks in Africa, especially for faith-based healthcare organizations, and the varied opinions on the Emergency Preparedness and Response (EPR) capacity of health institutions in Ghana, our research answers the question: “What challenges arose from CHAG's implementation of strategies for disease surveillance and infection prevention and control during Marburg outbreaks in Ghana?

### 1.1 Literature review

The term “Emergency Preparedness and Response” (EPR) denotes the capacity of health systems to prevent, detect and respond to health threats (diseases) promptly and effectively [11]. It describes a framework for mapping potential hazards and mobilizing resources and capacity to support operations at the national and facility levels [12]. EPR is extensively explored in literature with similar outcomes for developing nations [[13], [14], [15]].

According to Obermeyer et al. [16], Africa is susceptible to disease resurgence because the institutional framework for EPR is weak and non-responsive. A review of Ghana's preparedness and response capacity against COVID-19 identified the country's weak testing capacity, human resource training gaps, inadequate isolation centres, and long turnaround time for sample processing, among others, as setbacks to the fight against COVID-19 [14]. Further, Afulani et al. [13] investigated the perceived preparedness and response capacity of 472 health workers in Ghana against COVID-19 using WHO and Centre for Disease Control and Prevention's (CDC) COVID-19 preparedness Tools and Guidelines for Healthcare Professionals and Facilities as the assessment standard and found concerning gaps for policy considerations. The study revealed that 72.3 % of health workers were not adequately prepared to handle hazards, 45.8 % lacked training needs, 75.4 % lacked PPEs, 30.3 % without isolation facilities; 15.9 % had no guidelines to report suspected cases, 61 % had poor communication strategies, and 34.3 % were ignorant about the protocols to follow to manage confirmed COVID -19 cases. Similarly, Asiedu-Berkoe et al. [17] investigated the state of Public Health Emergency Preparedness and Response capacity of Ghana and identified many gaps: Regarding Planning and Coordination before during and after public health emergencies, the paper found the overdependence on government funding and the lack of institutionalised standard operating procedure to be a major problem. Additionally, concerning the structure and functions of disease surveillance, the paper described the health system as dysfunctional with weak multisectoral coordination. Again, on Ghana's response and recovery capacity, the paper criticised the country's unstructured referral, sample management and transportation systems, and health institutions' dysfunctional rapid response, vector control, laboratory and testing, and monitoring and evaluation capacities as inimical to the effective management of major disease outbreaks.

Conversely, other publications applauded Ghana's EPR capacity in comparison with other African nations. For instance, while generally admitting the need to beef up the EPR capacity of Ghana's Health System, Sarkodie et al. [14] commended Ghana's health sector and national-level EPR approaches to COVID-19. They observed that at the health sector levels, the provision of adequate infrastructure, logistics, human resources, and engagement of key stakeholders through public education and sensitization campaigns, among others, enabled rapid implementation of protocols to contain the disease. In addition, there were press releases and media engagements to elicit their support, and training of health workers on surveillance and case identification, IPC, and the use of Personal Protective Equipment (PPE). At the national level, the EPR for public health emergencies were revised. At the same time, Public Health Emergency Management Committees (PHEMCs), the Rapid Response Teams equipped, and the National Technical Coordinating Committee (NTCC) and the Emergency Operations Centre (EOC) were activated. Next, a sensitive case definition was adopted using the Ebola Virus Disease form to reduce the likelihood of missing cases. Ghana's response plan was driven by data and science, society and government policies. Specifically, international flights were grounded, movements restricted (Imposition of Restriction Act 2020) [18], and Veterinary Services Department laboratory and other private laboratories were onboarded to complement the testing capacity of the Noguchi Memorial Institute for Medical Research (NMIMR) and the Kumasi Centre for Collaborative Research in Tropical Medicine (KCCR). On surveillance, there was a radical contact tracing protocol and the use of the digital thermometer to limit and stop community spread of the virus. In terms of risk communication, in addition to Presidential broadcasts, there were regular press briefings by the Ministry of Information and other social mobilization strategies through print and audio-visual media outlets.

Common to papers on Ghana's EPR is the recommendation for continuous evaluation of post-COVID-19 emergency preparedness at the facility level [14]; the evaluation of facilities' enforcement of regulatory frameworks [19], and ascertaining the status of Infection Prevention and Control (IPC) measures in health facilities [20]. These recommendations, while relevant to the drive towards health system strengthening in Ghana, say little about the capacity of private healthcare providers to respond to disease outbreaks. So far, none of the recorded literature has comprehensively explained the capacity of faith-based healthcare facilities in Ghana to respond to emergency outbreaks effectively [20]. As such, since faith-based health enterprises are major service providers and contribute at least 40 % share of the total public health service delivery, it is important to ascertain their emergency preparedness and response capacities [21].

Accordingly, with the recent MVD outbreak in a Ghanaian faith-based facility, this study explored the IPC and surveillance strategies adopted by the Christian Health Association of Ghana (CHAG) and the challenges they encountered. The WHO Disease 2019 (COVID-19) Strategic Preparedness and Response Plan (SPRP) for the WHO African Region (February 1, 2021–January 31, 2022) [9] was used to assess the achievements and shortfalls of disease surveillance and IPC measures implemented during the outbreak of MVD.

## 2 Materials and method

WHO Coronavirus Disease 2019 (COVID-19) Strategic Preparedness and Response Plan for the African Region (February 1, 2021–January 31, 2022) (SPRP-AFR) was adopted as the assessment standard to examine Ghana's response to MVD outbreak [9]. The research is a phenomenological study [22].

SPRP-AFR [9] is a regional framework for a holistic public health response to COVID-19 at the sub-national, national, and regional levels. The framework was designed to reflect the epidemiological changes and regional contexts of vaccination in Africa, highlighting the criticalness of sustained inter-agency response to the pandemic, providing adequate resources and building the capacity of African nations to save lives, suppress transmission, and mitigate the adverse effects of the pandemic on families, economies and health systems on the continent, and charting a roadmap against future outbreaks. There are eleven public health response pillars of WHO COVID-19 SPRP-AFR [9], built into a matrix. They comprise (i) Coordination, planning, financing and monitoring (CPFM), (ii) Risk communication, community engagement and infodemic management (RCCE-I), (iii) Surveillance, outbreak investigation and calibration of public health and social measures (SOPHS), (iv) Points of entry, international travel and transport, and mass gatherings (PIM), (v) Laboratories and diagnostics (LD), (vi) Infection prevention and control and protection of the health workforce (IPCP), (vii) Case management, clinical operations and therapeutics (CMCT), (viii) Operational support and logistics, and supply chains (OLS), (ix) Strengthening essential health services and systems (SEHSS), (x) Vaccination (V), and (xi) Research, innovation and evidence (RIE) [9]. Our study is focused on two of these pillars, namely, SOPHS and IPCP, and the scope of the research limited to the facility level. This enabled the study to be conducted within a predetermined budget.

### 2.1 Selection of participants and data collection

Faith-based healthcare organizations (FBOs) are religiously influenced charity institutions with clear goals of delivering healthcare to the underserved [23,24]. This research was undertaken in the Christian Health Association of Ghana (CHAG), the largest faith-based healthcare provider in Ghana and key player in the country's fight against Ebola, COVID-19 and MVD [21]. CHAG is also the largest local implementation partner of the Ghana Health Service and the second largest health service provider in Ghana. It operates as a semi-autonomous not-for-profit health service provider, guided by policies and regulations of the Ministry of Health, and dependent on it for its human resources. In addition to internally generated funds, CHAG facilities receive regular premium payments from the National Health Insurance Authority to finance their operations. However, since Churches are the owners of CHAG facilities, they bear the ultimate responsibility to buy medical equipment and finance their operations. CHAG is managed by 34 Church denominations with 34,589 employees and 374 facilities delivering primary, secondary, and tertiary healthcare services. CHAG currently serves about 11,308,640 Ghanaians [21]. Similar to other West African countries like Nigeria, FBOs are classified and managed the same way as government hospitals. However, whereas the Nigerian system is largely based on the cash-and-carry system, over 80 % of patients who receive healthcare services from FBOs in Ghana are covered by the National Health Insurance scheme. Given that FBOs played critical roles in the fight against Ebola and COVID-19, a study on the preparedness and response capacity of CHAG, the focal agency in the fight against MVD outbreak in Ghana, is timely.

The target population comprised clinical and nonclinical healthcare professionals who were directly involved in the management of MVD outbreak in Ghana. We used purposive sampling technique to select health workers whose descriptions and experiences align with the research question. Authorisation was obtained from CHAG, and the participants contacted through phone calls to participate in the study. Participation was voluntary. We interviewed 15 participants using a semi-structured interview guide with open-ended questions to examine two pillars of the WHO COVID-19 SPRP 2022. The number of interviews conducted was determined by data saturation [22]. The participants include one District Deputy Chief Disease Control Officer, one District SNO Public Health, one Hospital Administrator, one Senior Health Service Administrator, one Senior Medical officer, one Medical Director, one Medical Doctor, one nurse manager, four nursing officers, one enrolled nurse, one biomedical scientist and one human resource manager. The guide had four sections, namely participants' Demographic characteristics Isolation and quarantine, and Collaborations. The tool was sectioned in line with the WHO COVID-19 SPRP 2022. The interviews were conducted virtually on Zoom in the English language between April 2023 to August 2023 and recorded. The video files were transcribed, cleaned, coded and analyzed with NVivo (version 14) [22]. To ensure that the work environment and colleagues did not influence participants’ responses, each participant was required to isolate himself/herself during the interviews. Where isolation was not possible, the participant was interviewed after working hours.

#### 2.1.1 Validity and reflexivity

Credibility and trust were built by connecting with health workers, cross-checking data (triangulation), and involving participants in verifying findings. As this strategy ensured the accuracy of information collected, so also participants' freedom to choose when and where interviews were conducted enabled them to speak freely. Engaging health workers in candid discussions about IPC measures and challenges strengthened the research validity. Further, recognizing their roles as CHAG employees, the researchers considered participants' potential anxiety. As such, the authors avoided discomforting questions. Furthermore, the authors ensured neutrality by concentrating solely on participants’ responses and avoiding descriptors that could identify them.

### 2.2 Data analysis

Braun and Clarke's [25] thematic analysis was used to thematize and analyse data. The researchers began by familiarizing themselves with the data, reading and re-reading transcripts to identify patterns and initial ideas. Next, initial codes were generated under relevant thematic areas of the WHO COVID-19 SPRP 2022, summarising similar ideas and systematically labelling meaningful segments of the data. These codes were then collated into potential themes, organizing similar codes into major themes. The themes were independently reviewed and refined by each author, ensuring they accurately represented the data. The authors then came together to discuss, define and name the final set of themes to provide clear labels for analysis. Finally, the researchers produced a comprehensive report, supported by illustrative quotes with coherent narratives to support the identified themes.

### 2.3 Ethical considerations

We obtained ethical clearance from the Ghana Health Service Institutional Review Board with the review number GHS-ERC 007/02/23. The participants consented to participate in the study. To protect participants' identity, all identifiers, including the facility's name and place of residence, were either replaced with pseudonyms or not mentioned in the paper.

## 3 Findings

Table 1 shows the demographic characteristics of participants. These include a District Deputy Chief Disease Control Officer, a District SNO Public Health, a Hospital Administrator, a Senior Health Service Administrator, a Senior Medical officer, a Medical Director, a Medical Doctor, a nurse manager, 4 nursing officers, an enrolled nurse, one biomedical scientist and a human resource manager. There were more females (n = 9) in the study than males (n = 6). Also, the majority were aged between 30 and 39 years (n = 9), followed by those between 40 and 49 years (n = 4). Only two (n = 2) were between the ages of 20 and 29 years. Eleven participants were married at the time of the research and 12 were parents.

**Table 1:** Participant list.

NAME	DESIGNATION	AGE (YEARS)	GENDER	MARITAL STATUS	PARENTAL STATUS	YEARS OF EMPLOYMENT
DDCO	District Deputy Chief Disease Control Officer	44	M	Married	Yes	2
DPHN	District SNO, Public Health	36	F	Married	Yes	5
HA	Hospital Administrator	43	M	Married	Yes	14
SHSA	Senior Health Service Administrator	34	F	Married	Yes	5
SMO	Senior Medical Officer	37	F	Married	Yes	4
MDir	Medical Director	42	M	Married	Yes	16
MD	Medical Doctor	46	M	Married	Yes	10
NM	Nurse Manager	37	F	Married	No	4
NO.1	Nurses Officer	29	F	Single	Yes	7
NO.2		36	F	Single	Yes	10
NO.3		34	F	Married	Yes	8
NO 4		39	F	Married	Yes	8
EN	Enrolled Nurse	26	F	Single	No	3
BS	Biomedical Scientist	31	M	Single	No	4
HRM	Human Resource	38	M	Married	Yes	5

### 3.1 Infection prevention and control and protection of the health workforce (IPCP)

Coordination of Infection Prevention and Control measures is critical to the effective management of disease outbreaks. To understand this process, the participants were asked about protocols implemented to prevent the spread of MVD. Topics such as quarantine, evacuation, decontamination, and other safety protocols were examined (Table 2). The logistical capacity of the facility to manage these responsibilities was also assessed. Findings from the interviews show that participants generally commended the prompt quarantine of workers following the outbreak, but expressed concern about the evacuation and decontamination process, citing the lack of essential decontamination chemicals, ambulance and morgue.

**Table 2:** Emerging themes. Table 2Highlights the main theme and subthemes emerging from the interviews.

OBJECTIVES	THEMES	SUB-THEMES
Examine the emergency preparedness and response strategies for surveillance and IPC enforcement during MVD outbreak in Ghana.	IPCP	- Social distancing and quarantine protocols - Effectiveness of decontamination efforts - Logistical constraints of evacuation procedures - Strategies for preventing facility/community transmission
	SOPHS	- Community surveillance and engagement - Challenges of medical equipment and technologies - Risk management and screening - Training and capacity building

#### 3.1.1 Social distancing and quarantine protocols

The study explored the protective protocols enforced to prevent infections among workers and clients and the challenges encountered. The participants' responses indicated that the lack of intensive care units, isolation centres and dedicated housing units to quarantine exposed staff had significantly impeded the safeguarding effort. Some of them had this to say:

We didn’t have an ICU and Isolation unit. So when the outbreak was confirmed, one of the two emergency rooms with three beds was quickly turned into an isolation unit (NO 2).

At first, the hospital wanted to have one building dedicated to staff who came in contact with infected patients. But because the facility could not afford it, we decided that we would do home quarantine. So since most of us come from different places, we rented apartments. So, we were told not to travel and not go outside. The facility decided to offer the individuals who were quarantined some foodstuffs so that they would not go out to buy (MD).

Asking staff to go for self-isolation (quarantine) for 2 weeks became a challenge because we didn’t have the adequate number of staff. We had 2 prescribers at that time, and they were all exposed. Our PAs were also involved in the case so basically our OPD was going to be shut down. As a result, the Medical Director had a conversation with the Regional Director for assistance. Our Denominational Director also gave us 1 PA for locum and another PA who had done his national service here also informed us he was free so we engaged him for locum. So luckily our OPD was functioning. We also had one doctor we had sponsored to train in China who returned from his horsemanship awaiting posting, so he also came to support us (SHSA).

We had a washing area at the security end and clients were given masks at the point of entry. And from time to time, we walk through those who are sitting in the waiting area. If they are not masked up, we ask them to wear their mask (SHSA).

#### 3.1.2 Effectiveness of decontamination efforts

The participants described the decontaminating process used, and the challenges encountered. Some explained the significance of the exercise, noting that it ensured the safety of medical wards. Despite this, the participants lamented the lack of needed chemicals and protective materials like gloves, PPEs and BP apparatus to facilitate the decontamination effort. Some of them responded as follows:

The IPC team came around to inspect and do the necessary decontamination process and followed it up with training on IPC protocols (SHSA).

We were given chlorine solution to disinfect all our surroundings and when a car brings a patient to the facility, once the patient comes out of the car, we disinfect the car that brought the patient to prevent infections … But we were not wearing gloves, so it opened the door to infections. As a result, we always disinfect our knobs, chairs, and desks … We were given PPEs to wear when attending to the patients, but the PPEs were not enough (NO 2).

The BP apparatus we used, the bedspread and other things, we soaked them in chlorine solution for hours before washing them (EN).

#### 3.1.3 Logistical constraints of evacuation procedures

All participants considered the timely evacuation of dead bodies an important IPC measure. For proper evacuation to be carried out, participants called for well-resourced morgues, ambulances, and washing areas, among others. Unfortunately, the facility lacked most of these infrastructures. Some of them expressed their frustration as follows:

We don’t have a morgue here and the family was told not to attend to the body because we suspect a viral disease (DPHN).

Concerning evacuating bodies, our clients are deeply enculturated. And so understanding people is a critical issue for us. The first case, for instance, came from the north so the moment the patient expired, they took the body to the north. Later on, I think some of the relatives got infected and died. They took the body to the north so we couldn’t manage it well but the second one was what we were able to manage well. We talked with the family a bit so they sent it home and the district director followed it up to ensure they had disposed of it very well. From our side too, because of the lack of morgue and ambulances, it took at least 24 hours to evacuate the first body from the facility (MD).

#### 3.1.4 Strategies for preventing facility/community transmission

The study explored strategies for restricting access to infectious areas, contact tracing, screening, and workshops aimed at reducing transmissibility. The findings show that most of the hospital staff were engaged in these activities. However, given the absence of essential resources and personnel, the interventions were not effective. When asked to describe the facility's systems for prevention, detection, and response to disease outbreaks, some of them had this to say:

The general care and maternity care ward, including theatre, everything, was put on hold … until the disease control guys were called to spray these areas (SMO).

My role was to sensitize the nurses in the facility on how they are supposed to protect themselves, and what they are expected to look out for in walk-in clients and during community outreach. I also sensitize them on the types of cases they can refer to the facility (DPHN).

Our orderlies have a set of scrubs they wear when they come to work, they don’t use their attires from work and send them home. The set of equipment Orderlies used in cleaning the lab is different from what they use at the emergency unit. We don’t let them use the same things from other wards in the lab. So, the emergency unit has its set of equipment used for cleaning and other things (BS).

After the confirmation, the relatives were contacted for screening … So they contacted all those who came into contact with the deceased … My director and the disease control officer were mostly going to houses or calling them on the phone to ask them how they were feeling and if they were experiencing signs and symptoms. They use phone calls because, at a point, the relatives didn’t want them (the medical team) to visit them with our vehicles because they thought people would stigmatize them … the hunters, farmers and those who deal in bush meat were also involved in risk communication so we went to all the sub-district and brought them to one particular point and we explained the Marburg disease and how they are supposed to handle the meat ….We usually take samples for lab investigation to confirm whether they’ve also been infected (DPHN).

### 3.2 Surveillance, outbreak investigation and calibration of public health and social measures (SOPHS)

The study explored the surveillance and trend-monitoring systems used during the outbreak. According to the participants, protocols such as case identification in the medical facility, community surveillance, and laboratory testing for case identification, among others, were implemented (Table 2). These activities were completed concurrently with other public health and social measures at the community and facility levels. Some of the participants explained this as follows:

#### 3.2.1 Community surveillance and engagement

Given the importance of social measures during health emergencies, the research explored obstacles faced by public health officers when handling samples and dead bodies in communities to prevent transmission. The District Public Health Nurse narrated this as follows:

The family was told not to attend to the body because we suspected a viral disease. Not knowing the client was a Muslim, so they went for the body before the MVD confirmation. After the confirmation, the relatives were contacted and samples taken from all those who came into contact with the deceased … We were supposed to get some employees to go into the communities regularly. But we didn’t do it because we did not have the ZB funds to fund visits by the directorate’s disease control unit (DPHN).

#### 3.2.2 Challenges of medical equipment and technologies

Availability of medical equipment is critical to response efforts against health emergencies. Despite this, the research participants complained about the inadequacy of essential technologies and tools to conduct surveillance, triage and test samples. Two nurses and the medical director had these to say:

We have only one monitor to screen patients who come around. Also, we don’t have enough thermometers. Again, when the samples are taken, the disease control officers do not come for them early. The biomedical scientist stores them in the refrigerator until the next day so they can pick them up for investigation (NO 2).

Things like PPEs, ICU and isolation centres, ambulances, morgue, and even some tools or instruments like BP apparatus, ventilators, and other things are either lacking or inadequate, and this is a challenge to the facility (MD).

The facility needs an x-ray machine to detect respiratory tract infections … we had a few testing kits but even those testing kits came in late … On the ambulance system, because we don’t have one of our own, we always call the national ambulance if there’s a referral to other facilities. When we call them, they respond to us, but often it delays (NM).

#### 3.2.3 Risk management and screening

Given the level of exposure among staff of the hospital, participants were asked to describe the measures implemented to prevent the spread of MVD in the facility. The Medical Director and the Senior Medical Officer explained this as follows:

We classified the exposure into low risk, moderate risk, and high risk. Those of us who performed procedures on the patients were classified under the high-risk group and most of us were prescribers. In our facility, prescribers are housed in the hospital premises, so, it was easy for us to monitor our movement during quarantine. The only challenge will be those who were at low risk and isolated in their own homes. We have people who were monitoring them. They went to their homes and neighbourhoods to monitor them. For contact tracing, I will even score it like 99% or 100%, because we went regularly to the neighbourhoods of these two detected cases to see their relatives. This was done almost every day for 3 weeks because the incubation period was two weeks. The contact tracing was excellent (SMO).

Management paid occasional visits to make sure they did not disobey quarantine orders. Aside from unannounced visits, there were regular calls to find out how they were feeling and monitor the experience of symptoms (MD).

It was the district disease control team and the biomedical scientists that we regularly worked with in sending samples. We also have working relationship with other health facilities in the district. We call on them when we have one or two challenges to deal with … Also, facility has a positive working relationship with GHS, NCHS, CHAG, the MoH, and the director of nursing at MoH. The National Public Health department also came and they made us prepare a logistic request through the district so they brought us some items like PPEs (HA).

#### 3.2.4 Training and capacity building

The research explored the adequacy and effectiveness of training and capacity-building exercises for staff in the facility. This was done to assess the measures used to transmit knowledge and skills to workers during the outbreak. When asked to describe the training methods used and challenges encountered, some of the participants had these to say:

We didn’t have any IPC training tools on Marburg, so we just did standard and transmission-based precautions. At the lab, we did safe injection practices and techniques, and healthcare waste management for our orderlies. We had to take them through how to handle waste that comes through the Isolation room and training on environmental cleaning control Processes, handling used instruments, proper placement of patients and training on the use of PPEs (BS).

We called the disease control officer and his team to have a discussion with the medical staff to train them on case definitions and identification of symptoms. The District Director has been helping us in all activities, he comes regularly for meetings and discussions, and if there are challenges, he helps us and I go there for meetings. I present my public health issues to him (HA).

We had a short workshop on how to wear the PPEs. But we lacked staff during that time because we were 15 staff for the unit, seven of us went to quarantine, so the nurse manager had to go and bring staff from other units to help at emergency unit (NO 2).

## 4 Discussion

We adopted two pillars of the WHO COVID-19 SPRP-AFR (2022) to assess the emergency response capacity of faith-based facilities for Surveillance, Outbreak Investigation, and Calibration of Public Health and Social Measures (SOPHS), and for Infection Prevention and Control and Protection (IPCP) protocols during the outbreak of Marburg Virus Disease (Table 2).

### 4.1 Infection prevention and control and protection of the health workforce (IPCP)

IPCP enables the early identification and management of patients infected with infectious diseases to prevent transmission to staff, transmissions among staff, and transmissions between staff and clients, among others. The pillar promotes community health and awareness measures, including capacity assessment of ICUs, training for clinical and non-clinical staff, social distancing, hand hygiene, and the availability of personal protective equipment (PPE), among others [9].

The coordination of infection prevention and control (IPC) measures emerged as a critical aspect of effective disease outbreak management, with participants highlighting protocols related to quarantine, evacuation, decontamination, and safety measures [26]. The suspension of general wards, maternity services, and theatres during the outbreaks of Marburg highlights the prioritization of IPC measures. However, the cessation of routine services also underscores the strain on overall healthcare delivery, signaling potential long-term consequences on the health workforce and community health.

While commendation was expressed for prompt quarantine measures, a noteworthy concern was the absence of essential decontamination chemicals. The challenges in obtaining these chemicals may signify a broader systemic issue, potentially hindering the immediate response capacity of CHAG during outbreaks, leaving the facility vulnerable to disease spread. Challenges in the decontamination process, such as the lack of gloves during disinfection procedures, pose ongoing risks of infection transmission within CHAG facilities [27]. This vulnerability highlights the importance of consistent availability and utilization of protective equipment to mitigate potential outbreaks caused by lapses in IPC protocols. In addition, consistent with observations from outbreaks of COVID-19, Ebola and MVD in Nigeria, Kenya, and Tanzania, among others, the study found cultural practices and belief systems to be a hindrance to preparedness and response efforts during health emergencies [[28], [29], [30]]. The cultural practices of clients, as seen in the reluctance to retain bodies for proper management in Ghana, for instance, pose significant hurdles. Instances, where bodies were taken to distant locations due to cultural practices, underscored the challenges in managing infection spread and proper disposal. The lack of resources like morgues and ambulances prolonged evacuation periods, potentially amplifying public health risks and compromising timely containment of outbreaks [27,31]. These challenges point towards the necessity of community engagement and strengthened infrastructure for timely and safe evacuation procedures.

Social distancing and quarantining measures were implemented, yet the insufficiency of resources, such as personal protective equipment (PPE), emerged as a recurring concern. The establishment of isolation units and the provision of personal protective equipment (PPE) were crucial steps taken by the facility. However, unlike Tanzania, where lessons from COVID-19 led to the establishment of screening and isolation centres to control future outbreaks, the CHAG facility in Ghana, where MVD was detected, did not have an isolation center prior to the virus detection [28]. Further, the insufficiency of PPEs, as observed in health systems across Africa [32], raises questions about the preparedness and resource allocation for IPC. The shortage of PPEs hindered healthcare workers' ability to safely attend to patients, amplifying the risk of exposure [33]. This scarcity could lead to increased vulnerability of healthcare workers during future outbreaks, potentially impacting the continuity of essential healthcare services. Similarly, the shortage of staff and subsequent challenges in enforcing self-isolation underscored the vulnerability of FBOs in managing outbreaks. This observation matched reports from Guinea where deploying staff to combat Ebola and COVID-19 led to a shortage of critical workforce to respond to MVD [29]. In Ghana, collaborations with regional and denominational directors to secure additional staff demonstrated a resourceful approach but also hinted at the need for broader support mechanisms. Addressing staff shortages during self-isolation (quarantine) therefore highlights another critical gap in CHAGs' IPC capabilities. The limited number of prescribers and staff exposed to cases strained operational capacity, prompting the need for external assistance [9]. While temporary staff support and regional interventions managed to sustain operations, the fundamental issue of inadequate staffing for outbreak scenarios remains unresolved, posing a recurrent challenge for CHAG.

In addition, home quarantine measures, necessitated by financial constraints, led to logistical challenges for staff members residing in different locations [9]. While the decision to provide foodstuffs and conduct regular checks exhibited adaptability, it also exposed the limitations in facility resources and the potential strain on the affected individuals. The responses imply that the effectiveness of IPC measures is intricately tied to the financial and logistical capacities of CHAG, emphasizing the importance of external assistance during outbreaks. Yet, reliance on external residences for quarantine likewise raised concerns about equitable access to safe and adequate living conditions during future outbreaks, potentially exacerbating disparities among staff members [34]. Given the lack of alternative sources of support, the participants emphasized the need for external support in disease control efforts, mentioning assistance from internal stakeholders for decontamination and collaboration with disease control authorities.

In conclusion, the data indicates that the capacity of faith-based organizations for infection prevention and control is influenced by multiple factors, including resource availability, logistical capabilities, and external support. The challenges identified in IPC protocols, staff shortages, financial constraints, and cultural considerations may impact the ability of the CHAG to manage disease outbreaks effectively [31]. Addressing these challenges requires a comprehensive approach involving enhanced resource allocation, collaborative partnerships, and continuous training to strengthen CHAG's overall capacity to protect healthcare workers and communities during infectious disease crises. The identified gaps in infection prevention and control within CHAG facilities during MVD outbreak call for multifaceted interventions to address shortages in essential resources such as PPEs and decontamination chemicals which are crucial to fortify the frontline defense against infectious diseases. Investment in infrastructure, including morgue facilities and ambulance services, is imperative to streamline evacuation processes and minimize delays in managing deceased patients. Moreover, capacity-building initiatives focusing on training and educating staff regarding infectious disease protocols and surveillance systems are essential. Strengthening internal resources and establishing collaborations with external agencies for technical support could bolster the resilience of these organizations in responding to future outbreaks.

### 4.2 Surveillance, outbreak investigation and calibration of public health and social measures (SOPHS)

Surveillance, outbreak investigation and calibration of public health and social measures is the third pillar of the framework. It monitors the detection, trend monitoring calibration of relevant public health measures to stop outbreaks. As presented in Table 2, the testing, quarantine and isolation of infected cases, treatment, contact tracing and early warning capabilities of the facility, and the health system at the national and sub-national levels were assessed [9,35].

The participants in our study employed a variety of surveillance and trend-monitoring systems during the outbreak, encompassing case identification in medical facilities, community surveillance, and laboratory testing. Simultaneously, public health and social measures were implemented at both community and facility levels. However, several challenges were revealed that hindered the effectiveness of these systems. Financial constraints emerged as a significant barrier, hindering regular community visits by disease control units and delaying the retrieval of samples for investigation [12]. Insufficient resources, including inadequate staffing, monitoring equipment, and testing kits, compromised CHAGs' capacity to respond effectively. This inadequacy, as highlighted by one participant and confirmed by observations from response effort against MVD in Guinea [29], Kenya [36] and Uganda [37], extends to crucial medical equipment, impacting the facility's ability to manage cases efficiently [9]. Another participant highlighted the absence of a disease control officer in their facility, impacting the regularity of community visits for active surveillance due to a lack of funding [37,38]. This gap in staffing and resources raises concerns about the facility's capacity to promptly detect and respond to potential outbreaks in the future [39].

Moreover, the data highlights commendable efforts in contact tracing and quarantine management within CHAG, particularly in high-risk exposure scenarios. The meticulous tracking of cases and diligent monitoring of contacts demonstrate the potential for effective outbreak control. However, this success is juxtaposed with significant challenges faced in providing adequate training and resources for infection prevention and control (IPC). The inadequacy of essential resources emerged as a recurring theme, affecting various facets of public health and social measures. Participants reported shortages or inadequacies in personal protective equipment (PPE), ICU and isolation centres, ambulances, morgue facilities, and essential medical instruments [33]. The implications of these deficiencies are far-reaching, posing significant challenges to the overall preparedness and response capacity of the facility [12]. The absence of specialized training tools for specific diseases like Marburg exposes a critical gap in preparedness, necessitating tailored training modules and protocols to address emerging threats comprehensively [39]. Given the evidence of similar challenges in Equatorial Guinea, Nkfusai et al. [40] recommended collaboration between Africa Centres for Disease Control and Prevention (Africa CDC), World Health Organization and member states to prevent health emergencies and the spread of infectious diseases.

Another critical issue pertains to the inadequacy of screening resources and delays in sample collection. Much like Uganda [41], insufficient monitoring infrastructure, such as a shortage of thermometers and a single monitor to screen patients, was reported in this study [28,32]. Additionally, delays in the collection of samples for investigation due to the unavailability of disease control officers were noted. These deficiencies in the surveillance process raise questions about the timely identification and isolation of potential cases, potentially contributing to the spread of infections within the community and healthcare settings [12]. The absence of critical equipment like x-ray machines and delayed procurement of testing kits directly impairs the rapid identification and management of infectious diseases. The reliance on national ambulances for patient transfers poses logistical challenges and emphasizes the urgency for establishing robust in-house transportation systems. Reliance on national ambulances introduces delays in response times during referrals to other facilities. These gaps not only impede timely interventions but also highlight a potential bottleneck in the coordination of public health measures, as prompt response and transfer of patients are critical in controlling the spread of infectious diseases [39].

Moving forward, bridging these gaps necessitates a multifaceted approach. CHAG require dedicated funding streams to support regular surveillance activities, bolster staffing, procure necessary equipment, and invest in continuous training on disease-specific IPC measures. Establishing partnerships with governmental agencies and fostering collaborations within the public health sector can alleviate resource constraints and enhance CHAG's response capacities. Additionally, implementing standardized protocols and specialized training modules tailored to emerging infectious diseases will fortify preparedness and ensure a more effective response to future outbreaks.

In conclusion, the findings reveal significant gaps in the capacity of CHAG for surveillance, outbreak investigation, and the calibration of public health and social measures. The deficiencies in staffing, resources, and training impede the effectiveness of these systems. Addressing these gaps requires strategic investments in human resources, essential medical supplies, and targeted training programmes. Moreover, establishing internal capacities for surveillance and response is crucial to reduce reliance on external services and enhance the overall resilience of faith-based organizations in managing public health emergencies.

### 4.3 Limitations

The study has inherent limitations. Interviews were conducted one year post the MVD outbreak in Ghana, potentially leading to recall bias and forgetfulness. To mitigate this, we provided a brief background summary to each interviewee before posing substantive questions. Instances of social desirability surfaced initially, with some staff misinterpreting the study as a performance appraisal of their unit during the outbreak. To address this, we paused interviews, clarified the research purpose, and reiterated the questions. Additionally, our study did not explore all eleven pillars of SPRP-AFR (2021), crucial for assessing the complete emergency response and control capacity of health systems. Future investigations should therefore consider delving into these aspects. Then again, our study's scope was confined to a single health service agency, limiting generalizability. As such, we recommend further research on the cross-national emergency response capacities of health systems in Africa and other LMICs.

## 5 Conclusion

The research examined the effective coordination of SOPHS and IPCP measures in CHAG facilities during the Marburg Disease outbreak in Ghana.

Findings from the study underscored the importance of strengthening IPC protocols in faith-based healthcare institutions. For instance, enforcement of IPC protocols unveiled significant progress but also exposed critical deficiencies in resources and staffing within CHAG facilities. Similarly, the assessment of SOPHS measures implemented brought to light gaps in staffing, testing and laboratory resources, and training, hindering the effectiveness of health systems in responding to disease outbreaks.

To tackle these challenges, CHAG and owners of faith-based healthcare facilities must augment resource allocation by fostering collaborative partnerships and providing continuous training to enhance overall IPC and SOPHS capacities. Collaborations and partnerships with public health authorities are emphasized to alleviate resource constraints and bolster SOPHS capacities in CHAG institutions. Strategic investments in human resources, essential medical supplies, and targeted training programs are recommended. These initiatives would strengthen the resilience of faith-based healthcare institutions against health emergencies.

## Data availability

No. Data will be made available on request.

## Funding statement

This research did not receive funding.

## Additional information

No additional information is available for this paper.

## CRediT authorship contribution statement

**Herman Nuake Kofi Agboh:** Writing – review & editing, Writing – original draft, Methodology, Investigation, Formal analysis, Data curation, Conceptualization. **George Adjeisah Adjei:** Writing – review & editing, Validation, Supervision, Software, Project administration, Formal analysis, Conceptualization. **Grace Adjei-Okai:** Writing – review & editing, Writing – original draft, Methodology, Formal analysis, Data curation, Conceptualization. **Caroline Awotwe:** Validation, Software, Project administration, Data curation. **Benjamin Martey Ossom:** Writing – review & editing, Software, Project administration, Data curation. **Lily Yarney:** Writing – review & editing, Validation, Supervision, Methodology, Conceptualization.

## Declaration of competing interest

The authors declare that they have no known competing financial interests or personal relationships that could have appeared to influence the work reported in this paper.
