# Supplementary material for: Infection prevention and control: Qualitative study of the preparedness and response of Christian health Association of Ghana to Marburg virus disease in Ghana
Source: Heliyon. 2024 May 25;10(11):e31953. doi: 10.1016/j.heliyon.2024.e31953 (PMC11176786; doi:10.1016/j.heliyon.2024.e31953)
Supplement: Multimedia component 1 [file mmc1.docx]

**SUPPLEMENTARY INFORMATION**

**Table 1** **Participant List**

Table 1 details the demographic characteristics of clinical and nonclinical health workers who were interviewed for this research.

| NAME | DESIGNATION | AGE (YEARS) | GENDER | MARITAL STATUS | PARENTAL STATUS | YEARS OF EMPLOYMENT |
| --- | --- | --- | --- | --- | --- | --- |
| DDCO | District Deputy Chief Disease Control Officer | 44 | M | Married | Yes | 2 |
| DPHN | District SNO, Public Health | 36 | F | Married | Yes | 5 |
| HA | Hospital Administrator | 43 | M | Married | Yes | 14 |
| SHSA | Senior Health Service Administrator | 34 | F | Married | Yes | 5 |
| SMO | Senior Medical Officer | 37 | F | Married | Yes | 4 |
| MDir | Medical Director | 42 | M | Married | Yes | 16 |
| MD | Medical Doctor | 46 | M | Married | Yes | 10 |
| NM | Nurse Manager | 37 | F | Married | No | 4 |
| NO.1 | Nurses Officer | 29 | F | Single | Yes | 7 |
| NO.2 |  | 36 | F | Single | Yes | 10 |
| NO.3 |  | 34 | F | Married | Yes | 8 |
| BO 4 |  | 39 | F | Married | Yes | 8 |
| EN | Enrolled Nurse | 26 | F | Single | No | 3 |
| BS | Biomedical Scientist | 31 | M | Single | No | 4 |
| HRM | Human Resource | 38 | M | Married | Yes | 5 |

Source: Field data, 2023

**Table 2 Emerging themes**

Table 2 highlights the main theme and subthemes emerging from the interviews.

| OBJECTIVES | THEMES | SUB-THEMES |
| --- | --- | --- |
| Examine the emergency preparedness and response strategies for surveillance and IPC enforcement during MVD outbreak in Ghana. | IPCP | - Social distancing and quarantine protocols - Effectiveness of decontamination efforts - Logistical constraints of evacuation procedures - Strategies for preventing facility/community transmission |
|  | SOPHS | - Community surveillance and engagement - Challenges of medical equipment and technologies - Risk management and screening - Training and capacity building |

Source: Field data, 2023

**Interview Guide**

Infection Prevention and Control: Qualitative Study of the preparedness and response of Christian Health Association of Ghana to Marburg Virus Disease in Ghana

**Demographics**

- Age
- Gender
- Marital Status
- Parental Status
- Profession
- Years of employment
- Current designation

| Key Area | Questions |
| --- | --- |
| Isolation and Quarantine | 1. When the Marburg disease was detected in your facility, how did your everyday medical practice and lifestyle change?   *Prompts*:   - What new measures were put in place in response to the detection of the disease? - Did you have access to appropriate types and quantities of PPE? - What personal lifestyle changes have you made?  1. How many days were you quarantined? 2. Describe your concerns about the effectiveness of the quarantine. 3. What are some coping methods or things you do to maintain or boost your mental health during disease outbreaks? What did you find worked or didn’t work? |
| Emergency Preparedness and response | STRUCTURAL AND NON-STRUCTURAL SAFETY   1. Describe your facility’s critical systems for the prevention, detection and response to epidemic-prone hazards in your facility 2. Describe the types of equipment/devices and supplies in your facility to effectively prevent, detect and respond to disease outbreaks? 3. How adequate are they – describe each piece of equipment mentioned. 4. How does your facility’s location and role in the district health system predispose it to imminent disease outbreaks? 5. How secure were your hospital wards to manage the Marburg outbreak?   DETECTION AND EMERGENCY RESPONSE   1. How adequate was your facility’s human resource capacity to respond to the Marburg disease? (Which critical human resources did you lack?) 2. What kind of training did you (your staff) undergo to adequately respond to the outbreak? 3. What safety and support services did you have in place to assist infected/exposed staff? 4. Describe the adequacy of your facility’s logistical capacity to respond to the Marburg disease (Which logistics did you lack? – Lab, ambulance, isolation centres, ICU, ventilators, PEs, testing kits, etc.) 5. Describe if any, the financial challenges you encountered during:    1. Detection of Marburg    2. Response 6. How do you collect and manage data on disease outbreaks? What challenges do you face when collecting, managing and/or transferring data for testing/analysis? 7. How did you share/transfer critical information during the outbreak of the Marburg disease? 8. Describe the evacuation, decontamination and safety procedures adopted during the outbreak of Marburg disease. |
| Collaboration | 1. Briefly describe the relationship between your organization and the district health system. 2. How is the partnership managed administratively? 3. To what extent does the district health system take your organization’s opinions seriously when decisions are to be made? 4. Under what circumstances do you rely on informal personal relationships or procedures when certain things need to be done? 5. What different types of information and knowledge do you share with your district health system? 6. Does your organization have problems getting in touch with the district health system when you need to contact them? If so, how are such problems resolved? 7. In what ways has your district health system positively influenced the management and control of Marburg Virus Disease? 8. What have been some of the drawbacks for your organization in working with district health system? |
